# Correlation between Volumes Determined by Echocardiography and Cardiac MRI in Controls and Atrial Fibrillation Patients

**DOI:** 10.3390/life11121362

**Published:** 2021-12-08

**Authors:** Simona Manole, Claudia Budurea, Sorin Pop, Alin M. Iliescu, Cristiana A. Ciortea, Stefania D. Iancu, Loredana Popa, Mihaela Coman, László Szabó, Vasile Coman, Zoltán Bálint

**Affiliations:** 1 IMOGEN Research Institute, County Clinical Emergency Hospital, 400006 Cluj-Napoca, Romania; simona.manole@gmail.com (S.M.); claudiabudurea@yahoo.com (C.B.); popsorin98@gmail.com (S.P.); alin_m_iliescu@yahoo.com (A.M.I.); cristianaciortea@yahoo.com (C.A.C.); stefania.iancu@ubbcluj.ro (S.D.I.); paplory@yahoo.com (L.P.); mihaela_c87@yahoo.com (M.C.); szabolaci81@gmail.com (L.S.); vasile.coman@gmail.com (V.C.); 2 Faculty of Medicine, “Iuliu Hatieganu” University of Medicine and Pharmacy, 400012 Cluj-Napoca, Romania; 3 Faculty of Physics, Babeș-Bolyai University, 400084 Cluj-Napoca, Romania; 4 Institute of Life Sciences, University of Agricultural Sciences and Veterinary Medicine Cluj-Napoca, 400372 Cluj-Napoca, Romania

**Keywords:** left atrial volume, left ventricular volumes, echocardiography, cardiac MRI, atrial fibrillation

## Abstract

Aims: We aimed to compare cardiac volumes measured with echocardiography (echo) and cardiac magnetic resonance imaging (MRI) in a mixed cohort of healthy controls (controls) and patients with atrial fibrillation (AF). Materials and methods: In total, 123 subjects were included in our study; 99 full datasets were analyzed. All the participants underwent clinical evaluation, EKG, echo, and cardiac MRI acquisition. Participants with full clinical data were grouped into 63 AF patients and 36 controls for calculation of left atrial volume (LA Vol) and 51 AF patients and 30 controls for calculation of left ventricular end-diastolic volume (LV EDV), end-systolic volume (ESV), and LV ejection fraction (LV EF). Results: No significant differences in LA Vol were observed (*p* > 0.05) when measured by either echo or MRI. However, echo provided significantly lower values for left ventricular volume (*p* < 0.0001). The echo LA Vol of all the subjects correlated well with that measured by MRI (Spearmen correlation coefficient r = 0.83, *p* < 0.0001). When comparing the two methods, significant positive correlations of EDV (all subjects: r = 0.55; Controls: r = 0.71; and AF patients: r = 0.51) and ESV (all subjects: r = 0.62; Controls: r = 0.47; and AF patients: r = 0.66) were found, with a negative bias for values determined using echo. For a subgroup of participants with ventricular volumes smaller than 49.50 mL, this bias was missing, thus in this case echocardiography could be used as an alternative for MRI. Conclusion: Good correlation and reduced bias were observed for LA Vol and EF determined by echo as compared to cardiac MRI in a mixed cohort of patients with AF and healthy volunteers. For the determination of volume values below 49.50 mL, an excellent correlation was observed between values obtained using echo and MRI, with comparatively reduced bias for the volumes determined by echo. Therefore, in certain cases, echocardiography could be used as a less expensive, less time-consuming, and contraindication free alternative to MRI for cardiac volume determination.

## 1. Introduction

Cardiac volumes and functions are landmark parameters used in the clinical diagnosis, prognosis, and treatment of cardiovascular disease [1,2,3,4,5]. The accurate assessment of cardiac parameters, such as left ventricular volume (LV), ejection fraction (EF) [1,2,4], and left atrial volume (LA Vol) [3,6], has important implications for the clinical management of patients suffering from common cardiac disorders, for example, atrial fibrillation (AF) and heart failure [7,8,9]. Several noninvasive techniques are available for quantifying these parameters, but the values generated by different methods are often statistically different and, therefore, not directly comparable [2,5,10].

Currently, two of the most frequently used techniques in this field are echocardiography and cardiovascular magnetic resonance imaging (MRI). For both methods, rigorous scientific recommendations to accurately quantify cardiac chamber parameters have been elaborated and made available to clinicians [11,12]. In clinical cardiology, echocardiography is the standard tool for noninvasive cardiovascular imaging, being relatively inexpensive, widely available, and without limiting contraindications nor exposure to radiation [13,14,15]. However, echocardiography can be highly operator-dependent, has a limited spatial resolution with no direct volumetric measurements being possible (i.e., an estimate using linear dimensions), and is difficult to use with patients that have motion or breathing issues [5,14,16]. By comparison, cardiovascular MRI is considered the gold standard for assessing heart volumes and function and for quantifying fibrosis [17], with comparatively better spatial resolution and more precise border definition [2,18], However, cardiac MRI has several drawbacks: it is more expensive, examinations are time-consuming, and it comes with contraindications for some patients (e.g., marked obesity, claustrophobia, and metallic implants) [10,19].

In terms of direct comparisons of cardiac parameter values obtained using echocardiography with those obtained by cardiac MRI, numerous earlier studies have found that echocardiography tends to underestimate cardiac volumes as compared to MRI [20,21,22,23,24,25,26,27,28,29,30,31]. However, there are several studies that have shown excellent volume correlations between the two techniques in animal models [32,33] and good correlations for LVEF assessments in human patients [23]. As previous studies showed that echocardiography provides systematically lower value estimates for cardiac volumes (as compared to MRI), our current study aimed to test these findings in a mixed cohort of AF patients (with and without fibrosis) and healthy controls, which have a large span of values for the assessed cardiac parameters. We also aimed to determine a range of values where echocardiography could possibly be used as a fast and reliable substitute for cardiac MRI.

## 2. Materials and Methods

Patients with atrial fibrillation (AF) and healthy volunteers were recruited to participate in our clinical study. Our study (clinicaltrials.gov: NCT03584126) complied with the Helsinki Declaration, obtained ethical approval from the local Ethics committee of the County Clinical Emergency Hospital of Cluj-Napoca (Nr. 20117/04.10.2016) and recruited participants between 2017 and 2020. All participants gave their written informed consent to participate in the study. Inclusion criteria were age between 20–80 years, weight 50–120 kg. For the included AF patients, a diagnosis of persistent, permanent, or paroxysmal AF, not more than three months from conversion with constant chronical medication and an optimal echocardiographic window was mandatory. Exclusion criteria for healthy volunteers were the presence of cardiovascular diseases, hypertension, or diabetes. Whereas, for AF patients, exclusion criteria were other known cardiac or chronic diseases; patients under other chronic anti-inflammatory, oncology or study treatment; contraindication for cardiac MRI: prosthesis, pace-maker, metallic particles, pregnancy, known allergies and altered renal function (creatinine clearance < 40 mL/min determined after Cockcroft-Gault). All the participants underwent full cardiologic evaluation including ECG and echocardiography.

### 2.1. Echocardiographic Acquisition

Complete echocardiographic evaluations were performed with a Philips Affiniti 50 ultrasound device with a Philips S4-2 Cardiac Sector Probe transducer. From the apical 4-chamber and 2-chamber acoustic windows, two-dimensional images of the LA and LV were traced to calculate end-diastolic and end-systolic volumes (EDV and ESV, respectively) using the ellipsoid model [34]. EF and end-systolic LA Vol were also calculated using the Auto 2D quantification method. After a pulse control, three successive cycles were chosen in the case of AF patients. Data were stored and analyzed with QLAB Advanced Quantification Software (v10.5, Philips, Andover, MA, USA). Examples of cardiac volume determinations are presented in Supplementary Figure S1.

### 2.2. MRI Acquisition

The subjects fulfilling the previously presented inclusion criteria were considered suitable for cardiac MRI measurements. All subjects were imaged with a 3T whole-body MRI system (3.0T Discovery MR750w General Electric MRI scanner) using a dedicated body coil for signal reception.

To avoid postprocessing differences between echocardiography and MRI techniques, the values for LA Vol, EDV, and ESV determined by MRI were calculated using the same method as for echocardiography, namely using the ellipsoid model [34]. LA dimensions were measured at the end of the systolic phase of the left ventricle (before the opening of the mitral valve) on 2-CH and 4-CH FIESTA cine images. The LA volume was measured by the bi-plane area-length method with manually drawn endocardial contours in 2-CH and 4-CH views with exclusion of left atrial appendage and pulmonary veins.

LVEF was measured by manual planimetry of the left ventricular endocardium in short axis cine images at end-systole and end-diastole. At the basal slice, the ventricular cavity was differentiated from the atrium by the presence of ventricular myocardium and was confirmed on a co-registered long-axis image. Papillary muscles were regarded as part of the ventricular cavity. These quantitative CMR measurements were performed by a junior radiologist (LP) and validated by a senior radiologist (SM). LV EF was manually calculated based on the EDV and ESV measurements, according to the formula:

(1) $$\mathrm{EF}=100\frac{\mathrm{EDV}-\mathrm{ESV}}{\mathrm{EDV}}$$

The MRI acquisitions were performed within 10 days of echocardiography and cardiac evaluation, with a mean of 4 days. Cardiac MRI required synchronous cardiac gating with EKG and breath-holding techniques to overcome motion artifacts. Following acquisition, data were stored and analyzed offline using the workstation of the instrument. The cardiac MRI protocol consisted of dark blood sequences (Black Blood SSFSE), FIESTA cine sequences (ALL FIESTA CINE AST—with slice thickness 8, frequency 128, flip angle 60 and 20 views per segment), and post-contrast sequences: rest perfusion (FGRE Time Course), angiography imaging (Aorta CEMRA Asset) and LGE/MDE images (2D MDE, 2D PSMDE—slice thickness 8, frequency 256, flip angle 20 and 24 views per segment) in three different planes: short axis, four-chamber and two-chamber view. The MDE/LGE sequences were acquired with a time delay of 10 min after gadolinium injection (Gadovist, 1 mmol/mL, Bayer AG), showing contrast between normal myocardium (dark) and abnormal myocardium (hyperenhancement). 3D HEART sequence (slice thickness 0.8, frequency 256, flip angle 15) was also acquired and used to assess coronary abnormalities.

The participants were monitored constantly during the examination, and no immediate adverse effects were reported. The results and all data were exported and stored as DICOM files.

### 2.3. Statistical Analysis

The normality of data distribution was tested using Anderson-Darling, D’Agostino Pearson, Shapiro-Wilk and Kolmogorov-Smirnov tests. The echocardiographic and MRI data did not pass the normality tests. Thus, the variables are represented as median and interquartile range (IQR). Age and BSA passed the normality tests, and they are presented as mean ± standard deviation (SD), together with their range. Comparisons between MRI and echocardiographic measurements of LV volumes, LA volumes, and EF were performed using non-parametric and paired Student’s *t*-test, Wilcoxon ranking. Correlation coefficients were calculated using non-parametric Spearman correlation. Differences between the gender distributions inside the groups were tested using Fisher’s exact test. *p* values provided are two-sided; *p* < 0.05 was considered statistically significant. Bland-Altman analysis was used to assess the agreement between echocardiography and MRI. The age- and body-surface area (BSA)-related differences between healthy controls and AF patients were tested using a non-parametric and unpaired Student’s *t*-test, (Mann-Whitney U test). Statistical analyses were performed using GraphPad Prism (Version 6.01. of GraphPad Software, Inc., La Jolla, CA, USA).

## 3. Results

**Figure 1:**
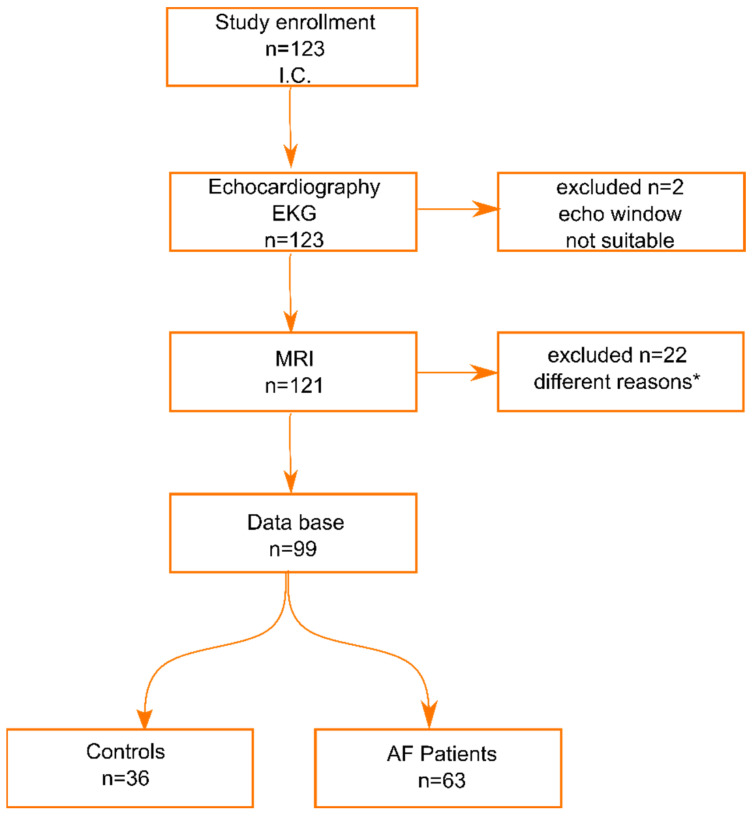
Subject enrolment diagram (I.C.—informed consent, * reasons explained in text).

In total, 123 subjects gave written informed consent to participate in this study. The study outline is presented in Figure 1.

Out of the total number, two patients were excluded because of poor quality or limited echocardiographic window. After general cardiologic evaluation and echocardiographic analysis, 121 participants were recruited for cardiac MRI measurements. Twenty-two subjects were excluded from the study/analysis for various reasons (seven subjects refused to undergo cardiac MRI; four procedures were abandoned due to symptoms of claustrophobia; two subjects had metal implants which made cardiac MRI impossible; one subject underwent undocumented neurosurgery; for two subjects, the measurements could not be made due to technical issues; and full echocardiographic LA Vol data were missing/could not be calculated for two Controls and four AF patients). After all procedures, 99 subjects were included in the database with full data. They were distributed into two groups: *n* = 63 AF patients and *n* = 36 healthy controls.

Table 1 presents the general characteristics of our study cohort. The number of subjects included in the LA Vol comparison was *n* = 99 (36 Controls and 63 AF patients).

For *n* = 6 Controls and *n* = 12 AF patients, full LV Vol data were missing due to data storage and archiving problems. Therefore, the cohort for LV Vol included in the study had a total of *n* = 81 subjects (30 Controls and 51 AF patients, Table 2).

**Figure 2:**
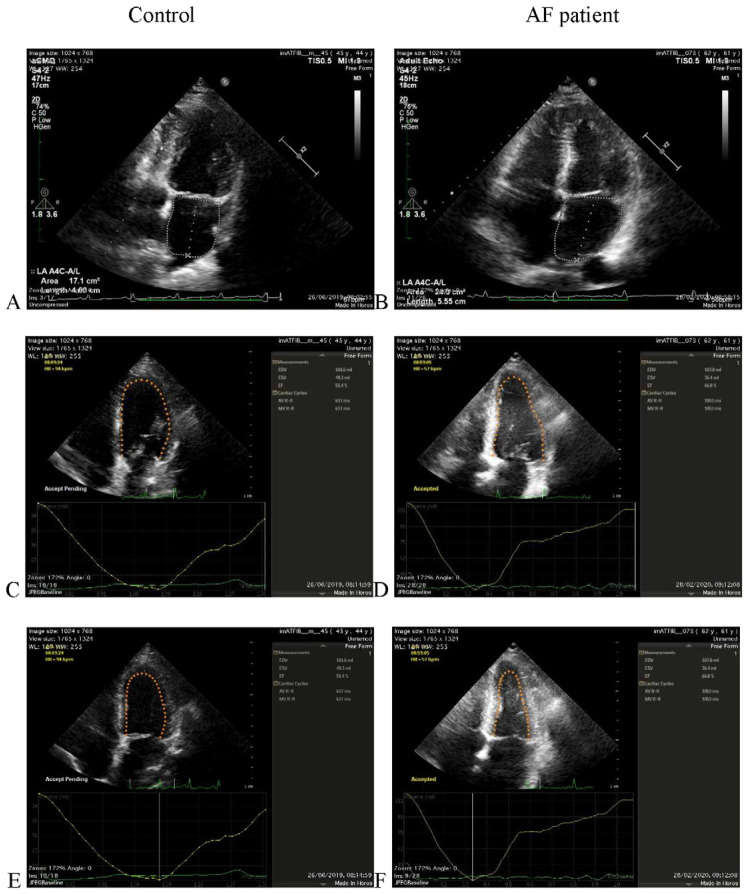
Representative echocardiography images from a healthy subject (Control—left panel) and from an AF patient (right panel) used to determine LA Vol (**A**,**B**), EDV (**C**,**D**), and ESV (**E**,**F**), respectively.

Figure 2 presents representative echocardiography images used to calculate the LA Vol, EDV, and ESV values for a healthy subject (Control) and for an AF patient.

**Figure 3:**
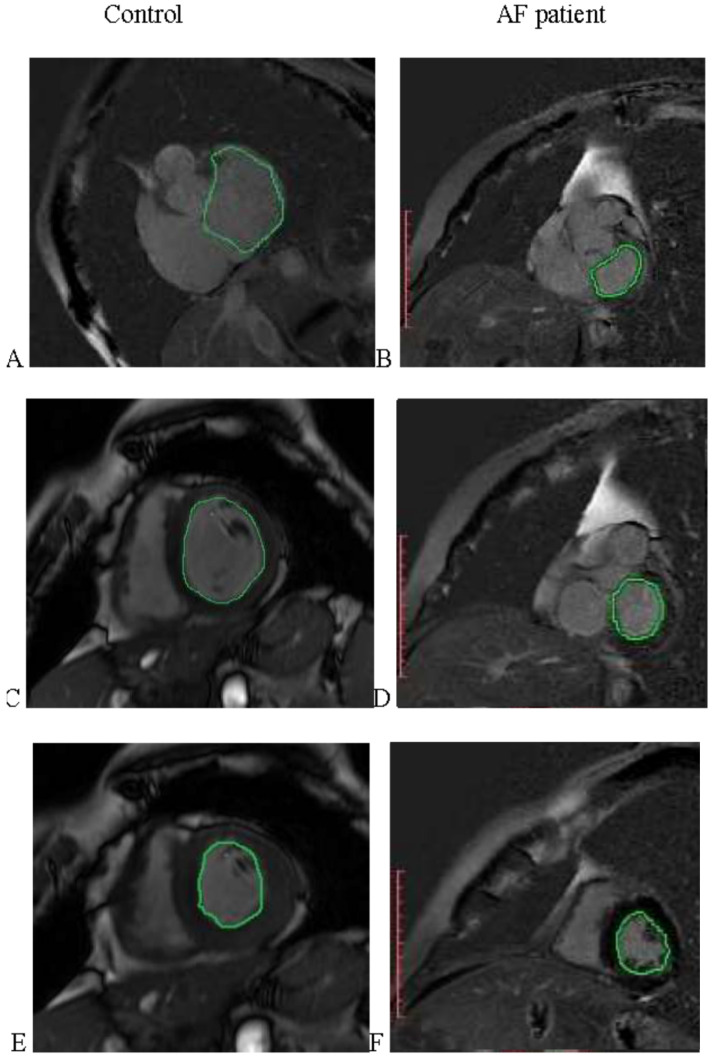
Representative cardiac MRI images from a healthy subject (Control—left panel) and from an AF patient (right panel) used to determine LA Vol (**A**,**B**), EDV (**C**,**D**), and ESV (**E**,**F**), respectively.

A series of representative cardiac MRI images are shown in Figure 3.

The comparison between echocardiographic and cardiac MRI measurements of LA Vol, EDV, ESV, and EF was assessed using the Mann–Whitney test. Table 3 presents the median and interquartile range (IQR) of the LA Vol, EDV, ESV, and EF examined by echocardiographic and cardiac MRI for all subjects, as well as for the Control and AF patient groups.

There was no significant difference between LA Vol measured by echocardiography and the values obtained by cardiac MRI (*p* > 0.05 for each group). However, the echocardiographic examination resulted in significantly lower values of the LV Vol than the cardiac MRI (*p* < 0.0001). The value of EDV for all subjects measured by echocardiography was 90.1 (77.65–102.5) mL, while the same parameter measured by cardiac MRI reached the value of 130.5 (113.9–160.6) mL. The same trend was observed for both groups: Control (94.75 (80.3–103.2) mL measured by echocardiography vs. 128.8 (106.8–159.9) mL measured by cardiac MRI) and AF patients (88.6 (77.4–100.9) mL measured by echocardiography vs. 135.6 (114.4–162.1) mL measured by cardiac MRI). The results were similar for ESV but presented smaller differences between the two techniques. The ESV value from echocardiography was 39.3 (30.95–44.55) mL for all subjects, 36.25 (28.4–41.65) mL for controls, and 36.3 (31.6–47.4) mL for AF patients, while the cardiac MRI resulted in mean values for ESV of 52.4 (36.9–69.7) mL for all subjects (*p* < 0.0001), 44.2 (35.1–54.7) mL for Controls (*p* = 0.0015), and 60.1 (44.31–76.7) mL for AF patients (*p* < 0.0001). Although the values for EF were similar for all subjects (*p* = 0.10) and the AF patients group (*p* = 0.93) regardless of the measurement technique, the values for the controls group measured by echocardiography were significantly lower (61.05 (57.63–66.7)%) than those from cardiac MRI (65.6 (61.9–71.6)%, *p* = 0.003).

**Figure 4:**
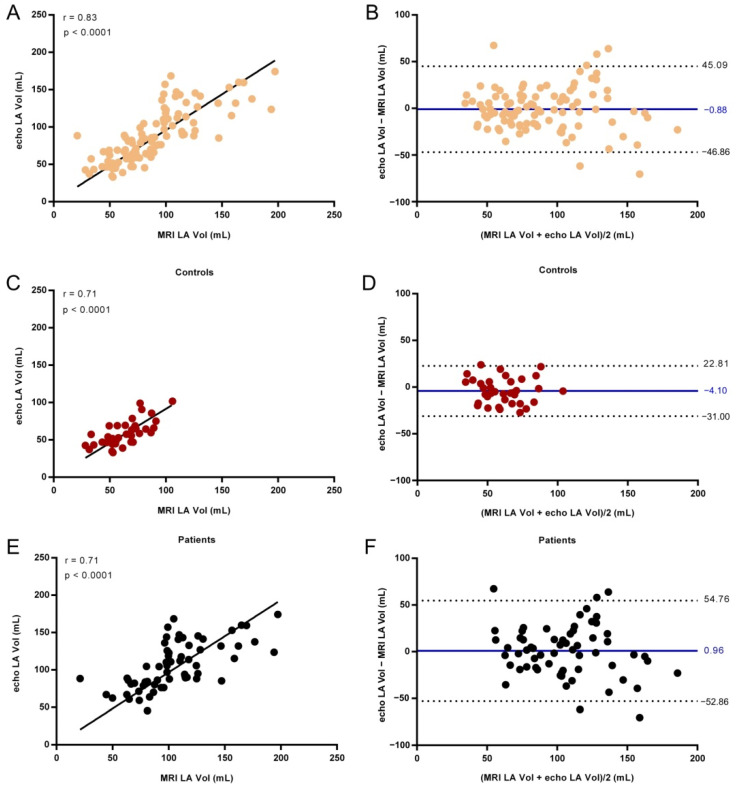
Left atrial volume (LA Vol) determined by echocardiography versus cardiac MRI examination for all subjects (**A**), Controls (**C**), and AF patients (**E**). The line shows the linear fit to the values. Spearman correlation coefficient r and the *p* value are presented in the plot. Bland–Altman analysis of the difference between MRI and echocardiographic measurements of LA Vol for all subjects (**B**), Controls (**D**), and AF patients (**F**). The solid line indicates the mean difference and the dashed lines the 95% confidence interval.

The strength of the relation between cardiac MRI and echocardiographic measurements of LA Vol, EDV, ESV, and EF was assessed using the Spearman correlation (Table 4) and the agreement between the two techniques was examined using Bland–Altman analysis (Figure 4).

Figure 4 presents the LA Vol values for all subjects, Controls, and AF patients measured by echocardiography plotted against the cardiac MRI values.

The echo LA Vol of all subjects correlated with the cardiac MRI LA Vol (r = 0.83, *p* < 0.0001), and the bias of the echo LA Vol measurements was close to 0 (−0.88 mL) with the 95% confidence interval (CI) of −46.86 to 45.09 mL. Good correlations between the echo LA Vol and cardiac MRI LA Vol values were observed both for Controls (r = 0.71 CI: 0.50–0.85, *p* < 0.0001) and AF patients (r = 0.71 CI: 0.56–0.82, *p* < 0.0001). The values with low bias were −4.10 mL for Controls with 95% CI: −31.00 to 22.81 mL and 0.96 mL for AF patients with 95% CI: −52.86 to 54.76 mL.

**Figure 5:**
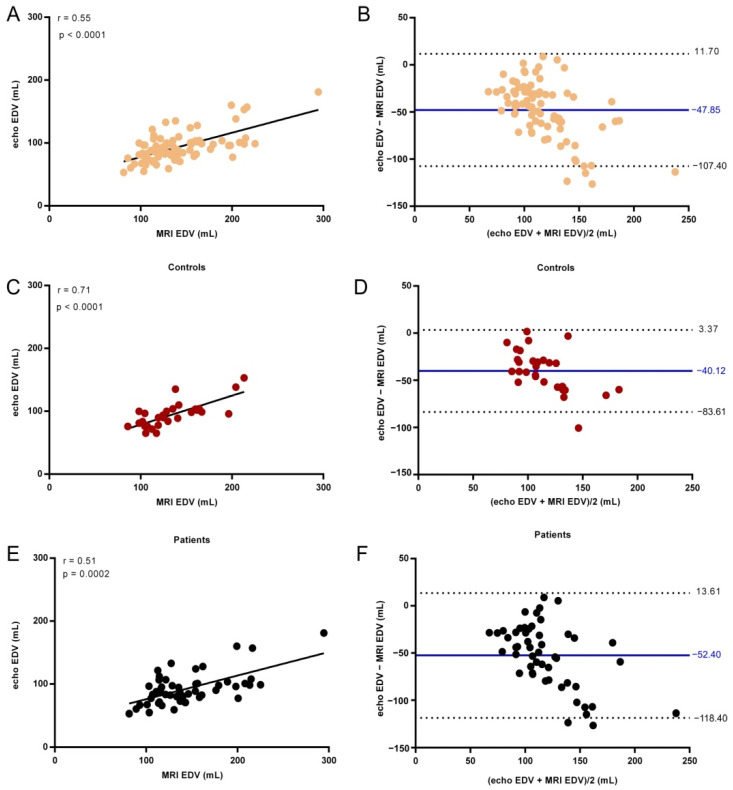
End-diastolic left ventricle volume (EDV) determined by echocardiography versus cardiac MRI examination for all subjects (**A**), Controls (**C**), and AF patients (**E**). The line shows the linear fit to the values. Spearman correlation coefficient r and the *p* value are presented in the plot. Bland–Altman analysis of the difference between MRI and echocardiographic measurements of EDV for all subjects (**B**), Controls (**D**), and AF patients (**F**). The solid line indicates the mean difference and the dashed lines the 95% confidence interval.

A significant positive correlation between echo EDV and cardiac MRI EDV was found for the Controls (r = 0.71, *p* < 0.0001), with a bias of −40.12 mL (Figure 5).

The correlation was weaker, though significant, for AF patients (r = 0.51, *p* = 0.0002) and all the subjects (r = 0.55, *p* < 0.0001). The obtained echocardiographic values were lower than the cardiac MRI values, with 52.40 mL for AF patients and 47.85 mL for all subjects. Although the echocardiographic values significantly correlated with cardiac MRI, a bias was present, with the latter resulting in higher values for EDV.

**Figure 6:**
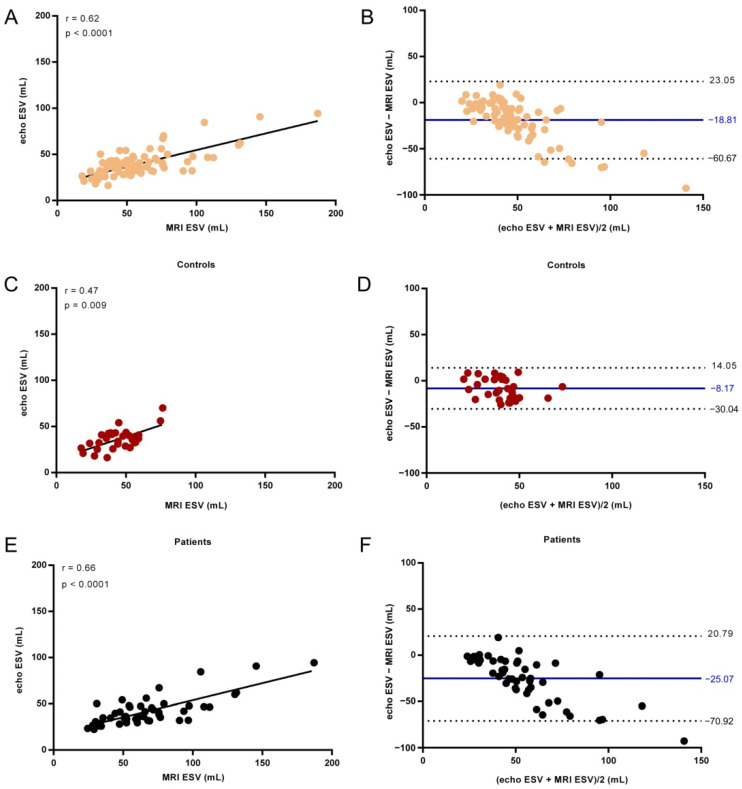
End-systolic left ventricle volume (ESV) determined by echocardiography versus MRI examination for all subjects (**A**), Controls (**C**), and AF patients (**E**). The line shows the linear fit to the values. Spearman correlation coefficient r and the *p* value are presented in the plot. Bland–Altman analysis of the difference between MRI and echocardiographic measurements of ESV for all subjects (**B**), Controls (**D**), and AF patients (**F**). The solid line indicates the mean difference and the dashed lines the 95% confidence interval.

Similarly, a significant correlation between echo ESV and cardiac MRI ESV was observed (Figure 6, r = 0.62 for all subjects, r = 0.47 for Controls and r = 0.66 for AF patients).

A negative bias was found for the comparison between echocardiography and MRI measurements of ESV for all subjects (bias of −18.81 mL), controls (bias of −8.17 mL), and AF patients (bias of −25.07 mL).

**Figure 7:**
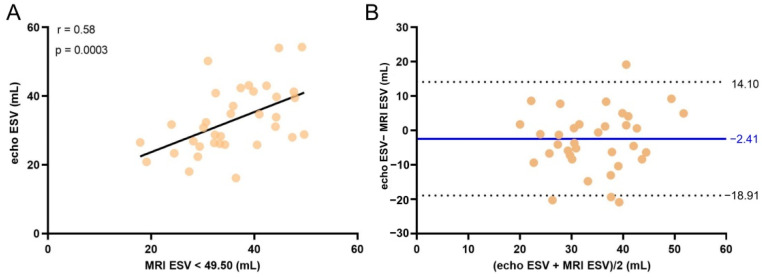
(**A**) End-systolic left ventricle volume (ESV) < 49.50 mL determined by MRI versus echocardiography examination for all subjects. The line shows the linear fit to the values. Spearman correlation coefficient r and the *p* value are presented in the plot. (**B**) Bland–Altman analysis of the difference between MRI and echocardiographic measurements of ESV for all subjects. The solid line indicates the mean difference and the dashed lines the 95% confidence interval.

We set an error threshold in measurements of 5% from the actual value, and we searched for the highest value of ESV for which echo measurements did not exceed this error. When we analyzed a subgroup of subjects with volumes smaller than a fixed value (in our case, 49.50 mL) there was good overlap between the two methods, a bias of −2.41 with −18.91 to 14.10 mL agreement limit (Figure 7). This threshold value was applied to all patients enrolled in the study, regardless of their cardiac condition (e.g., for Controls and for AF patients as well).

**Figure 8:**
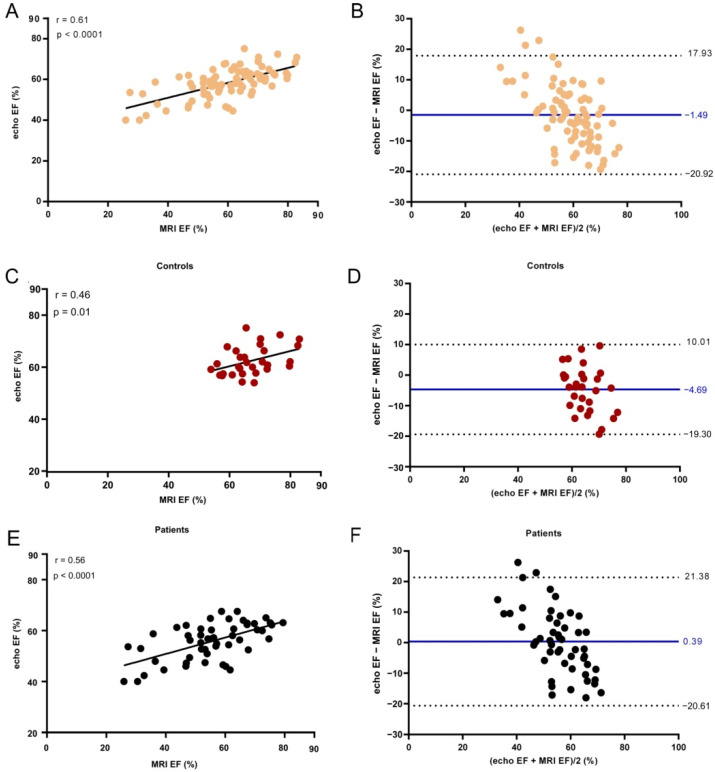
Ejection fraction (EF) determined by echocardiography versus cardiac MRI examination for all subjects (**A**), Controls (**C**), and AF patients (**E**). The line shows the linear fit to the values. Spearman correlation coefficient r and the *p* value are presented in the plot. Bland–Altman analysis of the difference between MRI and echocardiographic measurements of EF for all subjects (**B**), Controls (**D**), and AF patients (**F**). The solid line indicates the mean difference and the dashed lines the 95% confidence interval.

Echo EF significantly correlated with EF values determined by cardiac MRI (Figure 8). The Spearman correlation factor was r = 0.61 for all subjects. We found a constant underestimation of 1.49% (95% CI: −20.92% to 17.93%).

The same trend maintained for controls (r = 0.46 and *p* = 0.01) and AF patients (r = 0.56 and *p* < 0.0001). The underestimation of echo EF values reached a value of 4.69% (95% CI: −19.30% to 10.01%) for controls, whereas for AF patients, echocardiographic measurements showed higher values than cardiac MRI examination with a 0.39% positive bias (95% CI: −20.61% to 21.38%).

## 4. Discussion

Large values of LA Vol are a sign of chronicity in diastolic dysfunction, which is correlated with AF and stroke. A rapid and easy method for LA Vol determination is useful in decreasing the overall mortality after myocardial infarction [11,12]. In our study, the good correlation between LA Vol measurements by echocardiography and MRI (r = 0.83, *p* < 0.0001) and the low bias (−0.88) show the potential of echocardiography for measuring LA Vol. Significant correlation was observed both in Controls (r = 0.71, *p* < 0.0001) and AF patients (r = 0.71, *p* < 0.0001).

There was a small variability between the approximation methods used in echocardiography for volume measurements [35,36]. LA Vol measured using sphere and ellipsoidal approximations in 2D echocardiography and the disc summation method (Simpson’s rule) showed underestimations by 14–26% compared with MRI-measured LA Vol [37]. Our method based on global measurements showed an overall error of 20.52%, confirming the above results (please refer to Supplementary Table S1 for all individual values and Supplementary Figure S2 for the graphical representation of the value distribution).

As previously reported, the LV Vol is underestimated by echocardiography [11]. Similar results for EDV (bias of −33.3 mL) and ESV (bias of −16.2 mL) when comparing between values acquired by 2D echocardiography and MRI were found in healthy controls and patients suffering from different cardiac failures (e.g., myocardial infarction, hypertrophic cardiomyopathy, and patients with wall motion abnormalities) [23,38]. The underestimation of EDV (−52.4 mL) and ESV (−25.0 mL) by echocardiography when compared with MRI was also observed for AF patients (please refer to Supplementary Table S2 for all individual values and Supplementary Figure S3 for the graphical representation of the value distribution).

The bias and limits of agreement for LV Vol and LV EF were higher for the AF patients group compared to the Controls because of the assumption of ellipsoid geometry of the LV, which does not apply in a variety of cardiac pathologies [11]. It can be observed that for lower values of LV Vol, echocardiographic measurements were closer to the corresponding cardiac MRI results, but there was a tendency to underestimate for higher LV Vol values. This would explain the better overlap between the results obtained for ESV as compared to EDV (ESV has lower absolute values than EDV). The lower bias values for the controls group (−40.12 mL for EDV and −8.17 mL for ESV) compared to the AF patients group (−52.40 mL for EDV and −25.07 mL for ESV) also supports this observation.

A threshold for the correct determination of LV Vol measured by echocardiography should be researched. Our data suggest a threshold value of 49.50 mL. For ESV values lower than 49.5 mL (the threshold applied to the MRI values), echocardiography correlated with MRI (r = 0.54, *p* = 0.0007), and the bias was reduced from −18.81 mL for all values to −3.30 mL for ESV < 49.50 mL (Supplementary Figure S1). The mean relative value was significantly lower (20.50% for ESV < 49.50 mL compared to 30.72% for any value (Supplementary Table S2)). However, as EDV has generally higher values, echo and MRI turned out not to be interchangeable techniques for EDV determinations.

Echocardiographic determination of LVEF has previously been shown to offer good results when compared to the previous gold standard, radionuclide angiography [39], and with the current gold standard of MRI (EF bias of −0.66) [23]. A significant correlation with cardiac MRI results (r = 0.61, *p* < 0.0001) and a low negative bias was also observed in our study (bias of −1.49). The mean relative absolute error when compared with MRI reached a value of 14.90%. EF measured by echocardiography did correlate with the MRI values for healthy controls (r = 0.46 *p* = 0.01) and AF patients (r = 56, *p* < 0.001), with the mean differences between the two techniques being low in both groups (−4.69 for controls and 0.39 for patients).

Correlation between echocardiography and MRI measurements of cardiac volumes have previously been reported. Hoffmann et al. showed the necessity of contrast application in LV function measurements to lower the bias from 11 for unenhanced echo to 9.2 for enhanced echo [40]. Our results show a similar trend, but in our selected population of well characterized patients with AF and healthy controls, a lower bias was observed without any contrast application. Even if echocardiography does not fully reproduce the gold standard for cardiac volume acquisitions (MRI), its advantages (being a real-time technique, reduced costs, and reduced examination time) results in its recommendation for use in cardiac volume determinations for certain group of patients.

## 5. Limitations

Our results were based on a cohort of well-characterized AF patients and controls, as they were included in our clinical study after full cardiological evaluation and according to strict criteria (see Methods); therefore, our results cannot be extrapolated to other types of severe cardiac dysfunctions or genetic malformations.

## 6. Conclusions

A good correlation and reduced bias were observed for LA Vol and EF determined by echocardiography as compared to the gold standard, cardiac MRI, in patients with atrial fibrillation and healthy volunteers. A bias associated with larger LV Vol was observed. However, for volumes lower than 49.5 mL, echocardiography could be used as a less expensive, less time-consuming alternative for cardiac MRI in ESV volume determination. Moreover, this opens the possibility for LV volume determination for patients with MRI contraindications.

## Figures and Tables

**Table 1:** Subject characteristics for left atrial measurements cohort given as mean ± SD (range). Parameters with significant differences between Controls and AF patients are presented in italics. *p* < 0.05 was considered to indicate a statistically significant difference. Body surface area (BSA).

*N* = 99 Cohort for Left Atrial Measurements	Healthy Controls	AF Patients	*p* Value
No.	36	63	
Women/Men	26/10	41/22	0.51
Age	50 ± 8 (33–73)	63 ± 9 (38–78)	<0.0001
BSA	1.97 ± 0.25 (1.45–2.81)	1.98 ± 0.21 (1.39–2.56)	0.68

**Table 2:** Subject characteristics for left ventricle measurements cohort given as mean ± SD (range). *p* < 0.05 was considered to indicate a statistically significant difference. Body surface area (BSA).

*N* = 81 Cohort for Left Ventricle Measurements	Healthy Controls	AF Patients	*p* Value
No.	30	51	
Women/Men	21/9	35/16	0.99
Age	50 ± 8 (33–73)	63 ± 10 (38–78)	<0.0001
BSA	1.99 ± 0.25 (1.59–2.81)	1.98 ± 0.22 (1.39–2.56)	0.72

**Table 3:** Comparison between cardiac MRI and echocardiographic measurements of LA Volume, EDV, ESV, and EF of all subjects included in the study (all), Controls, and AF patients. Values are represented as median (IQR). *p* < 0.05 was considered to indicate a statistically significant difference.

	Cardiac MRI	Echocardiography	*p* Value
LA Vol (mL)			
All	86.8 (64.75–110.0)	85.3 (62.4–113.8)	0.54
Controls	64.3 (52.0–75.63)	57.5 (46.7–68.8)	0.085
AF patients	99.9 (81.05–124.6)	104.8 (83.8–132.0)	0.81
EDV (mL)			
All	130.5 (113.9–160.6)	90.1 (77.65–102.5)	<0.0001
Controls	128.8 (106.8–159.9)	94.75 (80.3–103.2)	<0.0001
AF patients	135.6 (114.4–162.1)	88.6(77.4–100.9)	<0.0001
ESV (mL)			
All	52.4 (36.9–69.7)	39.3 (30.95–44.55)	<0.0001
Controls	44.2 (35.1–54.7)	36.25 (28.4–41.65)	0.0015
AF patients	60.1 (44.31–76.7)	36.3 (31.6–47.4)	<0.0001
EF (%)			
All	61.3 (53.2–68.4)	59.1 (54.15–62.8)	0.10
Controls	65.6 (61.9–71.6)	61.05 (57.63–66.7)	0.003
AF patients	55.4 (47.5–64.85)	56.7 (50.9–62.0)	0.93

**Table 4:** Correlation between echocardiography and cardiac MRI measurements of LA Vol, EDV, ESV, and EF. *p* < 0.05 was considered to indicate statistically significant difference.

	Spearman r	*p* Value
LA Vol		
All	0.83	<0.0001
Controls	0.71	<0.0001
AF patients	0.71	<0.0001
EDV		
All	0.55	<0.0001
Controls	0.71	<0.0001
AF patients	0.51	0.0002
ESV		
All	0.62	<0.0001
Controls	0.47	0.009
AF patients	0.66	<0.0001
EF		
All	0.61	<0.0001
Controls	0.46	0.01
AF patients	0.56	<0.0001

## Data Availability

No publicly available datasets were used for this study.

## Supplementary Materials

The following are available online at https://www.mdpi.com/article/10.3390/life11121362/s1, Figure S1: Cardiac volume determination methods., Figure S2: The absolute error of the echocardiography estimation of LA Vol, EDV, ESV, and EF for AF Patients and Controls. Figure S3. Distribution and Bland-Altmann analysis results of the measured values based on participants’ gender (orange—female, green—male). Table S1: Individual Left atrium volume (LA Vol) values measured by MRI and echocardiography and their difference of the echocardiography estimations (%) for the 63 AF patients and 36 healthy Controls. Table S2: Individual EF, ESV, and EDV values measured by MRI and echocardiography and their absolute difference (%) of the echocardiography estimation for the 51 AF patients and the 30 healthy controls.
