# Supplementary material for: Correlation between Volumes Determined by Echocardiography and Cardiac MRI in Controls and Atrial Fibrillation Patients
Source: Life (Basel). 2021 Dec 8;11(12):1362. doi: 10.3390/life11121362 (PMC8707690; doi:10.3390/life11121362)
Supplement: Supplementary file 1 [file life-11-01362-s001.zip › life-1485228-supplementary.pdf]

life MDPI

Supplementary material of Correlation between Volumes Determined by Echocardiography and Cardiac MRI in Controls andAtrial Fibrillation Patients

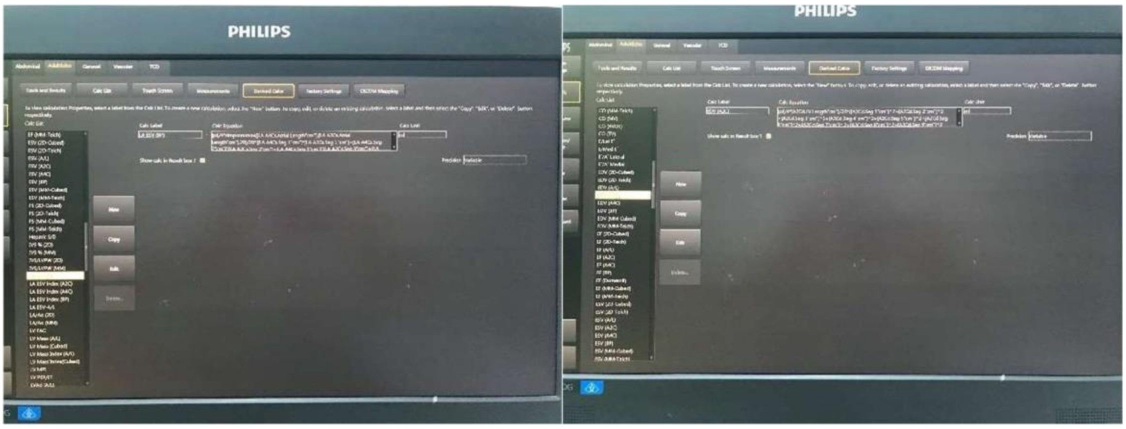

Figure S1. Cardiac volume determination methods.

Table S1. Individual Left atrium volume (LA Vol) values measured by MRIand echocardiography and their difference of the echocardiography estimations (%) for the 63 AF patients and 36 healthy Controls.

Control No.	MRI LA Vol (mL)	Echo LA Vol (mL)	Absolute diff. (%)
**1**	81.04	45.60	77.72
**2**	73.75	59.30	24.37
**3**	82.97	63.84	29.97
**4**	100.68	87.76	14.72
**5**	126.15	145.47	13.28
**6**	62.64	66.91	6.38
**7**	118.13	132.82	11.06
**8**	176.76	137.59	28.47
**9**	104.58	168.47	37.92
**10**	164.96	159.95	3.13
**11**	197.29	174.36	13.15
**Control No.**	**MRI LA Vol (mL)**	**Echo LA Vol (mL)**	**Absolute diff. (%)**
**12**	124.6	105.72	17.86
**13**	98.46	109.18	9.82
**14**	99.22	157.25	36.90
**15**	63.04	88.86	29.06
**16**	98.72	125.81	21.53
**17**	101.86	110.94	8.18
**18**	98.58	105.80	6.82
**19**	81.05	84.57	4.16
**20**	110.04	112.18	1.91
**21**	80.23	104.77	23.42
**22**	128.06	127.01	0.83
**23**	114.94	89.56	28.34
**24**	162.10	131.90	22.90
**25**	77.41	79.13	2.17
**26**	112.53	143.25	21.45
**27**	63.95	86.21	25.82
**28**	98.09	144.09	31.92

**29**	146.62	131.95	11.12
**30**	49.89	62.43	20.09
**31**	93.37	76.25	22.45
**32**	124.92	88.17	41.68
**33**	91.41	104.41	12.45
**34**	117.79	113.82	3.49
**35**	194.02	123.64	56.92
**36**	97.84	110.27	11.27
**37**	87.45	80.26	8.96
**38**	126.1	95.07	32.64
**39**	100.21	122.82	18.41
**Control No.**	**MRI LA Vol (mL)**	**Echo LA Vol (mL)**	**Absolute diff. (%)**
**40**	86.42	70.13	23.23
**41**	108.60	140.85	22.90
**42**	89.98	86.4	4.14
**43**	114.58	92.05	24.48
**44**	98.60	97.02	1.63
**45**	95.59	76.33	25.23
**46**	156.4	153.09	2.16
**47**	64.75	60.85	6.41
**48**	111.17	117.69	5.54
**49**	44.37	66.92	33.70
**50**	79.07	83.79	5.63
**51**	109.36	147.00	25.61
**52**	169.42	159.5	6.22
**53**	147.13	85.33	72.42
**54**	99.86	118.94	16.04
**55**	73.46	71.31	3.02
**56**	96.47	136.06	29.10
**57**	69.51	82.24	15.48
**58**	130.64	141.33	7.56
**59**	66.41	81.14	18.15
**60**	116.29	90.13	29.02
**61**	114.21	94.09	21.38
**62**	158.57	115.21	37.64
**63**	21.00	88.43	76.25

**Control No.**	**MRI LA Vol (mL)**	**Echo LA Vol (mL)**	**Absolute diff. (%)**
**1**	89.22	66.03	25.99
**2**	43.23	47.01	8.73
**3**	82.20	64.41	21.64
**4**	72.51	68.78	5.14
**5**	78.52	90.79	15.63
**6**	91.21	75.09	17.67
**7**	86.76	59.59	31.31
**8**	69.57	63.22	9.12
**9**	61.50	39.15	36.34
**10**	68.95	47.27	31.44
**11**	55.07	44.91	18.45
**12**	33.35	57.31	71.86
**13**	28.27	42.53	50.42
**14**	63.76	69.5	9.00
**15**	55.81	49.21	11.82
**16**	51.96	34.77	33.09
**17**	52.25	44.44	14.95
**18**	105.95	101.67	4.04

**19**	70.74	47.01	33.54
**20**	31.69	37.09	17.04
**21**	69.18	55.69	19.50
**22**	47.48	46.63	1.79
**23**	77.08	98.98	28.41
**24**	71.30	65.43	8.23
**25**	70.13	78.66	12.16
**26**	35.77	43.32	21.09
**Control No.**	**MRI LA Vol (mL)**	**Echo LA Vol (mL)**	**Absolute diff. (%)**
**27**	73.32	65.2	11.08
**28**	49.55	68.82	38.90
**29**	48.35	54.1	11.89
**30**	76.39	58.64	23.24
**31**	52.82	33.06	37.41
**32**	52.42	51.83	1.12
**33**	57.72	52.77	8.58
**34**	56.75	69.12	21.80
**35**	64.85	57.69	11.04
**36**	87.34	85.76	1.81

Table S2. Individual EF, ESV, and EDV values measured by MRI and echocardiography and their absolute difference (%) of the echocardiography estimation for the 51 AF patients and the 30 healthy controls.

Patient No.	MRIEF (%)	EchoEF (%)	Absolute diff. (%)	MRI ESV (mL)	Echo ESV (mL)	Absolute diff. (%)	MRI EDV (mL)	Echo EDV (mL)	Absolute diff. (%)
**1**	69.80	62.70	10.17	34.30	25.90	24.48	113.58	69.60	38.72
**2**	67.40	62.30	7.56	64.66	36.20	44.01	198.31	96.10	51.54
**3**	48.04	49.40	2.82	65.68	41.60	36.66	126.41	82.30	34.89
**4**	67.78	52.40	22.69	33.33	26.10	21.68	103.44	54.80	47.02
**5**	61.69	44.55	27.79	49.26	54.25	10.14	128.58	97.35	24.29
**6**	46.86	62.00	32.32	59.02	33.50	43.24	111.06	87.90	20.85
**7**	32.71	42.30	29.33	145.60	90.70	37.71	216.37	157.10	27.39
**8**	51.85	55.20	6.47	68.93	31.60	54.16	143.15	70.70	50.61
**9**	54.88	56.65	3.22	54.84	46.45	15.31	121.56	106.95	12.02
**10**	61.19	54.40	11.10	51.78	36.35	29.80	133.43	80.15	39.93
**11**	51.79	58.20	12.37	96.78	32.30	66.63	200.76	77.40	61.45
**12**	39.37	44.50	13.03	130.27	60.10	53.86	214.85	107.90	49.78
**13**	64.09	67.50	5.32	40.99	34.70	15.34	114.14	106.70	6.52
**14**	74.71	56.70	24.11	35.46	34.90	1.59	140.23	80.60	42.52
**15**	75.67	62.20	17.80	31.01	50.20	61.89	127.45	132.80	4.20
**16**	53.24	47.40	10.97	75.78	67.20	11.32	162.06	127.90	21.08
**17**	52.02	52.70	1.32	108.07	46.80	56.70	225.22	98.80	56.13
**18**	61.27	64.60	5.43	44.31	39.80	10.19	114.43	112.10	2.04
**19**	35.78	58.70	64.04	67.72	32.00	52.75	105.46	77.40	26.61
**20**	46.76	46.00	1.63	75.76	38.40	49.32	142.31	71.00	50.11
**21**	48.16	56.20	16.69	52.41	29.50	43.71	101.10	67.40	33.33
**22**	59.29	46.50	21.57	62.66	47.40	24.36	153.93	88.60	42.44
**23**	56.14	57.20	1.89	93.58	42.10	55.01	213.37	98.40	53.88
**Patient No.**	**MRI EF (%)**	**Echo EF (%)**	**Absolute diff. (%)**	**MRI ESV (mL)**	**Echo ESV (mL)**	**Absolute diff. (%)**	**MRI EDV (mL)**	**Echo EDV (mL)**	**Absolute diff. (%)**
**24**	73.71	65.00	11.82	24.47	23.40	4.39	93.11	66.70	28.36
**25**	53.94	50.90	5.64	60.12	29.65	50.68	130.52	59.15	54.68
**26**	67.35	62.90	6.61	29.06	22.40	22.91	89.00	60.30	32.25
**27**	58.76	67.50	14.88	47.37	28.00	40.89	114.84	86.20	24.94
**28**	43.73	61.20	39.96	90.55	32.00	64.66	160.91	82.40	48.79
**29**	31.60	52.90	67.41	70.53	45.50	35.49	103.12	96.70	6.22

**30**	36.45	47.90	31.41	187.16	94.40	49.56	294.51	181.00	38.54
**31**	79.47	63.10	20.60	28.17	26.90	4.52	137.23	72.90	46.88
**32**	57.13	54.80	4.07	75.63	40.80	46.05	176.40	90.10	48.92
**33**	30.50	40.00	31.13	131.44	62.10	52.75	189.14	103.60	45.23
**34**	55.40	60.20	8.67	51.60	36.30	29.65	115.69	91.20	21.17
**35**	62.49	58.00	7.19	59.42	33.10	44.30	158.42	78.80	50.26
**36**	47.53	58.00	22.02	76.74	35.40	53.87	146.27	84.30	42.37
**37**	55.04	64.80	17.73	52.53	33.50	36.23	116.84	95.20	18.52
**38**	64.85	56.30	13.19	47.67	41.30	13.36	135.63	94.50	30.33
**39**	62.87	60.60	3.61	50.84	34.60	31.94	136.91	87.70	35.95
**40**	54.19	56.70	4.64	71.57	43.60	39.08	156.21	100.80	35.47
**41**	53.22	52.60	1.17	97.42	47.80	50.94	208.26	100.90	51.55
**42**	66.06	64.00	3.12	60.88	35.20	42.18	179.38	97.80	45.48
**43**	25.92	40.00	54.35	79.29	50.00	36.94	107.02	83.30	22.16
**44**	56.85	53.85	5.28	66.56	56.10	15.71	154.25	124.15	19.51
**45**	51.81	60.60	16.96	54.30	47.90	11.78	112.69	121.60	7.91
**46**	60.25	45.90	23.82	32.38	28.70	11.37	81.46	53.10	34.82
**47**	75.16	63.00	16.18	30.14	30.80	2.19	121.33	83.40	31.26
**48**	46.95	47.20	0.54	105.75	84.60	20.00	199.33	160.20	19.63
**49**	27.33	53.60	96.10	112.17	46.40	58.63	154.36	100.10	35.15
**50**	72.48	59.90	17.36	32.26	26.40	18.18	117.24	65.90	43.79
**51**	70.80	60.30	14.83	33.47	28.30	15.45	114.61	71.30	37.79

**ControlNo.**	**MRI EF (%)**	**Echo EF (%)**	**Absolute diff. (%)**	**MRI ESV (mL)**	**Echo ESV (mL)**	**Absolute diff. (%)**	**MRI EDV (mL)**	**Echo EDV (mL)**	**Absolute diff. (%)**
**1**	70.83	62.00	12.47	47.89	39.50	17.51	164.15	103.80	36.77
**2**	64.93	63.80	1.75	54.56	35.60	34.76	155.61	98.40	36.76
**3**	63.37	59.50	6.11	74.84	56.10	25.04	204.31	138.40	32.26
**4**	79.72	60.40	24.23	32.51	40.90	25.82	160.26	103.30	35.54
**5**	76.64	72.40	5.53	27.38	18.00	34.27	117.23	65.20	44.38
**6**	79.93	62.10	22.31	23.94	31.70	32.40	119.31	77.90	34.71
**7**	63.05	60.10	4.68	44.13	31.10	29.53	119.44	89.90	24.73
**8**	68.14	54.00	20.76	39.85	41.40	3.89	125.09	89.90	28.13
**9**	57.63	56.70	1.62	44.26	33.90	23.40	104.46	76.40	26.86
**10**	65.67	61.80	5.89	35.92	37.10	3.29	104.62	96.70	7.57
**11**	64.30	57.40	10.73	30.65	32.40	5.73	85.83	75.90	11.57
**12**	59.29	67.80	14.35	52.90	27.10	48.77	129.95	84.10	35.28
**13**	53.91	59.10	9.62	59.21	40.80	31.10	128.48	99.80	22.33
**14**	63.53	63.80	0.43	59.04	36.90	37.50	161.88	101.80	37.11
**15**	70.24	70.90	0.95	49.67	28.80	42.01	166.86	98.90	40.73
**16**	64.15	54.30	15.36	76.41	70.00	8.39	213.18	153.20	28.14
**17**	60.90	57.00	6.41	54.97	38.20	30.51	140.61	88.80	36.85
**18**	82.48	68.30	17.20	17.87	26.50	48.25	102.05	83.70	17.98
**19**	55.90	61.30	9.66	56.97	32.80	42.42	129.18	84.60	34.51
**20**	70.08	68.80	1.82	29.40	25.30	13.96	98.26	81.10	17.46
**21**	67.57	60.00	11.21	44.78	54.00	20.59	138.09	135.00	2.24
**22**	83.00	70.80	14.70	19.11	20.90	9.36	112.41	71.70	36.22
**23**	71.38	66.30	7.12	56.21	32.40	42.36	196.43	95.90	51.18
**24**	65.49	75.10	14.67	36.45	16.20	55.55	105.62	65.10	38.36
**25**	62.17	66.20	6.48	40.56	25.80	36.39	107.22	76.40	28.75
**26**	72.52	60.80	16.16	38.96	43.10	10.64	141.75	109.90	22.47
**27**	68.65	57.70	15.95	50.08	43.60	12.94	159.77	103.10	35.47
**28**	56.83	56.90	0.13	42.39	43.00	1.43	98.20	99.90	1.74
**29**	57.92	57.40	0.89	52.34	39.90	23.76	124.37	93.60	24.74
**30**	72.37	59.20	18.19	37.37	42.40	13.45	135.24	103.90	23.17

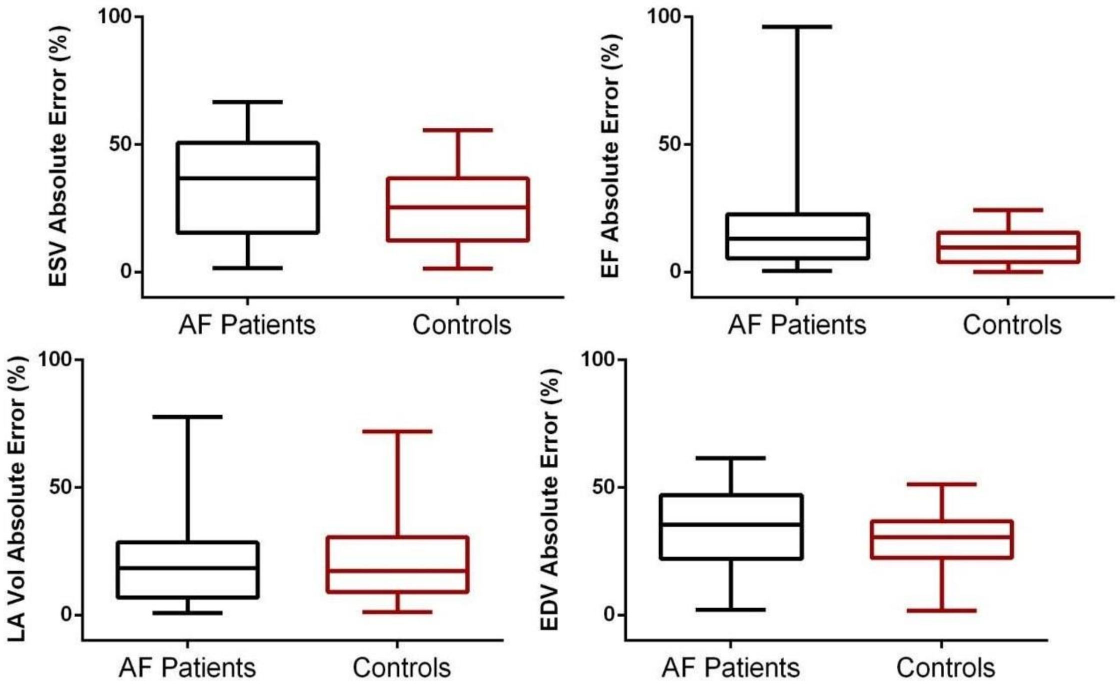

Figure S2. The absolute error of the echocardiography estimation of LA Vol,EDV, ESV,and EF for AF Patients and Controls.

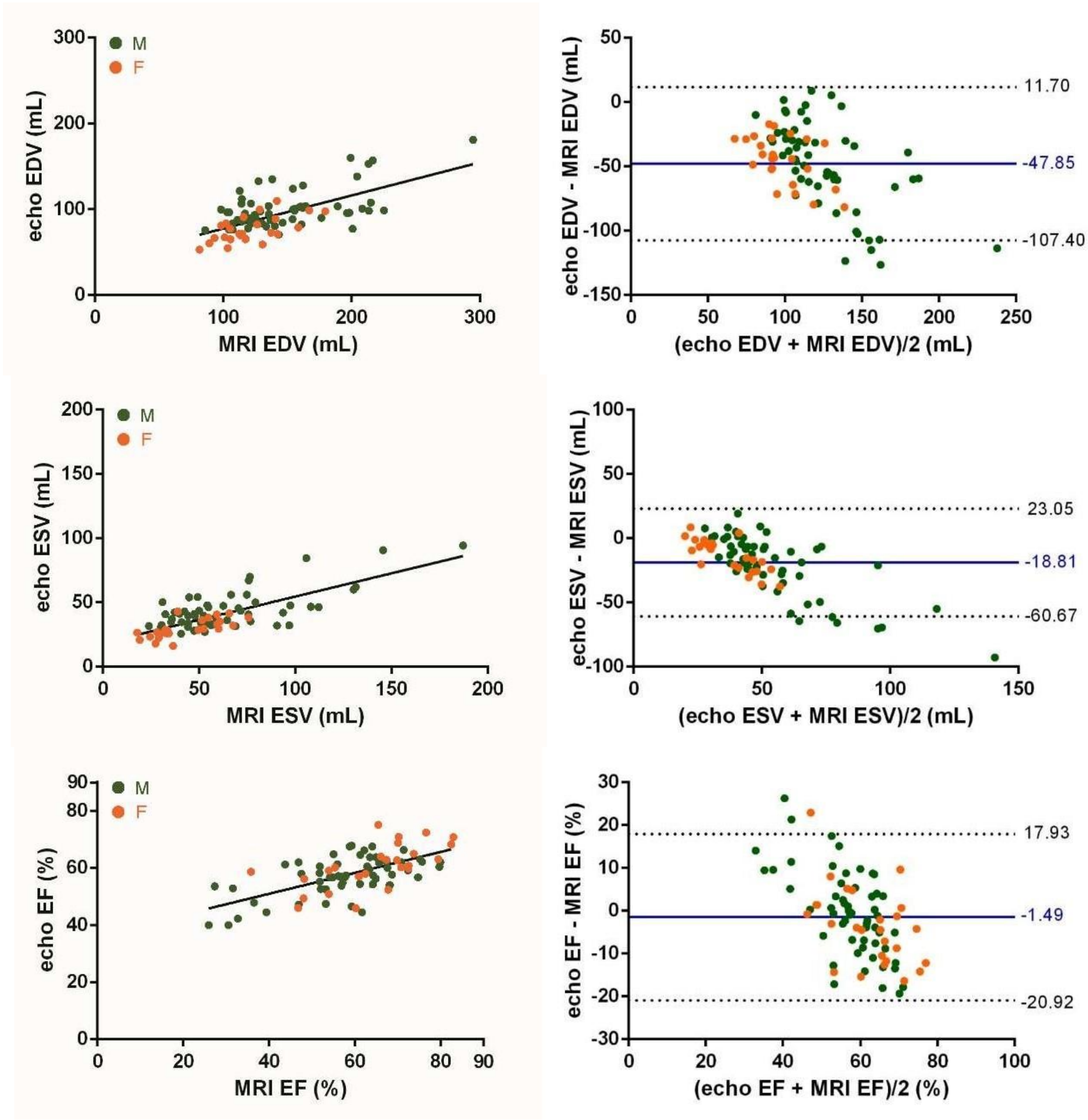

Figure S3. Distribution and Bland-Altmann analysis results of the measuredvalues based on participants' gender (orange-female, green-male).
